# Reliability and Responsiveness of a Novel Device to Evaluate Tongue Force

**DOI:** 10.3390/life13051192

**Published:** 2023-05-16

**Authors:** Marta Carlota Diaz-Saez, Alfonso Gil-Martínez, Inae Caroline Gadotti, Gonzalo Navarro-Fernández, Javier Gil-Castillo, Hector Beltran-Alacreu

**Affiliations:** 1 Physiotherapy Department, Centro Superior de Estudios Universitarios La Salle, Universidad Autónoma de Madrid, C/La Salle, 28023 Madrid, Spain; 2 CranioSPain Research Group, Centro Superior de Estudios Universitarios La Salle, Universidad Autónoma de Madrid, C/La Salle, 28023 Madrid, Spain; 3 Programa de Doctorado en Medicina y Cirugía, Universidad Autónoma de Madrid, C/Francisco Tomás y Valiente 5, 28049 Madrid, Spain; 4 Hospital Universitario La Paz-Carlos III. Institute for Health Research (IdiPaz), Paseo la Castellana, 261, 28046 Madrid, Spain; 5 Department of Physical Therapy, Nicole Wertheim College of Nursing and Health Sciences, Florida International University, 11865 SW 26th St Suite H3, Miami, FL 33199, USA; 6 Neural Rehabilitation Group, Cajal Institute, Spanish National Research Council (CSIC), Av. Doctor Arce, 37, 28002 Madrid, Spain; 7 Toledo Physiotherapy Research Group (GIFTO), Faculty of Physical Therapy and Nursing, Universidad de Castilla-La Mancha, Avenida de Carlos III s/n, 45071 Toledo, Spain

**Keywords:** feedback, muscle strength, neurofeedback, rehabilitation, reproducibility of results tongue

## Abstract

Background: Measurements of tongue force are important in clinical practice during both the diagnostic process and rehabilitation progress. It has been shown that patients with chronic temporomandibular disorders have less tongue strength than asymptomatic subjects. Currently, there are few devices to measure tongue force on the market, with different limitations. That is why a new device has been developed to overcome them. The objectives of the study were to determine the intra- and inter-rater reliability and the responsiveness of a new low-cost device to evaluate tongue force in an asymptomatic population. Materials and Methods: Two examiners assessed the maximal tongue force in 26 asymptomatic subjects using a developed prototype of an Arduino device. Each examiner performed a total of eight measurements of tongue force in each subject. Each tongue direction was measured twice (elevation, depression, right lateralization, and left lateralization) in order to test the intrarater reliability. Results: The intrarater reliability using the new device was excellent for the measurements of the tongue force for up (ICC > 0.94), down (ICC > 0.93) and right (ICC > 0.92) movements, and good for the left movement (ICC > 0.82). The SEM and MDC values were below 0.98 and 2.30, respectively, for the intrarater reliability analysis. Regarding the inter-rater reliability, the ICC was excellent for measuring the tongue up movements (ICC = 0.94), and good for all the others (down ICC = 0.83; right ICC = 0.87; and left ICC = 0.81). The SEM and MDC values were below 1.29 and 3.01, respectively, for the inter-rater reliability. Conclusions: This study showed a good-to-excellent intra- and inter-reliability and good responsiveness in the new device to measure different directions of tongue force in an asymptomatic population. This could be a new, more accessible tool to consider and add to the assessment and treatment of different clinical conditions in which a deficit in tongue force could be found.

## 1. Introduction

The tongue is a muscle that is part of the stomatognathic system and plays an important role in phonation, breathing, and eating [1,2]. The tongue has been classified as a muscular hydrostat structure due to its ability of movement and deformation without a bone holder preserving its volume [2]. The tongue performs five main movements: elevation, depression, protrusion, right lateralization, and left lateralization, and their multiple combinations. These movements are important during eating function for the intraoral manipulation of food and swallowing [3]. During chewing, the tongue muscles must coordinate with the masticatory muscles and the temporomandibular joints (TMJ). During breathing, tongue extrinsic muscles move the hyoid bone in the craniocaudal direction, allowing the pharynx to open. In addition, tongue muscles are coordinated with suprahyoid and infrahyoid muscles during swallowing and phonation. Therefore, muscle balance, understood as a phenomenon in which agonist and antagonist muscles work in coordination during voluntary movements or unexpected body perturbations, is important for tongue function. This phenomenon allows maintaining neural control of movements on a specific functional level and avoid excessive muscle control work. Likewise, it seems that coactivation could increase stiffness and movement speed, increasing the stability in kinematic chain cases. Thereby, any disturbance in muscle balance or dysfunction related to the tongue can lead to dysphagia, dysarthria or breathing difficulties [4].

Different studies have shown that the function of the tongue can be compromised in different clinical conditions such as oropharyngeal cancers [5], post-stroke sequalae [6], sequalae associated with Parkinson Disease [7], scleroderma [4], and chronic temporomandibular disorders (TMDs) [8]. For example, tongue weakness was shown to be associated with TMD patients with orofacial restricted mobility [9]. In addition, the aging process may also contribute to tongue disturbances. In this way, it has been reported that older adults have less tongue force than younger adults [10] and it is well known that deficient tongue force can compromise the behavior and efficiency of mastication and swallowing [11].

Due to the above-described factors, measurements of tongue force are important in clinical practice during both the diagnostic process and rehabilitation process [7,12,13]. Currently, there are few devices to measure tongue force on the market. The four most used and studied devices are the Kay Swallowing Workstation (KSW) [14], the Madison Oral Strengthening Therapeutic (MOST) [15], the Iowa Oral Performance Instrument (IOPI) [16], and the OroPress device [17]. The KSW (KayPENTAX Corporation, Lincoln Park, NJ, USA) device is a computerized system with three sensors which allow performing multiple simultaneous measurements in different tongue positions on the palate including pressure measurements during swallowing [18]. However, it is not portable due to its large size and it is very expensive. White et al. reported an excellent intrarater reliability in the KSW device in a healthy population (ICC = 0.92) [19]. The MOST device is a portable device with four or five sensors inside one intraoral piece with a small amount of pliable Reprosil Dental Putty (DENTSPLY International, York, PA, USA), which allows measurements of tongue isometric pressure against the hard palate in five different positions (anterior, middle, posterior, right, and left). The intraoral piece provides stability to the sensors, and it is easy to use by patients. The MOST device has not demonstrated its reliability and validity yet [20]. However, due to its price, it is less accessible for professionals and patients. Likewise, the IOPI (IOPI Northwest Company, LLC, Carnation, WA, USA) is the most used device in research because it is easy to use, it is portable, and has a silicon air-filled bulb, which allows measuring isometric tongue pressure against the hard palate. However, it has poor sensor stability which may cause measurement errors [21], and to date, there are no studies on validity and inter-rater reliability with the use of this device. Although a good inter-rater reliability to measure the maximum isometric tongue force with the IOPI was found (ICC > 0.75) [22], it has been shown to be less reliable than the other devices due to artifacts on the measurements [21]. Finally, the OroPress device is composed of a biomedical interface pressure transducer (BIPT-MS58 series, Measurement Specialities Ltd., Bevaix, Switzerland), an earpiece, and a wireless transmission module which transmits data to a remote laptop or notebook computer for real-time viewing and recording. This system measures the isometric and swallowing pressure applied by the tongue directly at the sensor tongue interface compared to those which apply the pressure indirectly through a column of air or fluid. It is characterized as being portable, having low-cost sensors, and being able to capture pressure while swallowing food or fluids. Oropress demonstrated good-to-excellent ICC values (ICC = 0.86) for its reliability [17]. Nevertheless, this device only has a pilot study trying to demonstrate its validity [17]. A bigger sample is needed to develop a good study of validity, and an intra- and inter-rater reliability study must also be carried out. This is very important to corroborate the safety, the psychometric properties, and the clinical utility of all these devices.

In order to overcome the limitations of the devices and improve the features, a validated, portable, handy, and lower-cost prototype device to measure tongue force was developed. The prototype has an intuitive interface, and it has been developed to assess and train tongue force in different movements, allowing its use not only for professionals, but also for patients for clinical and home rehabilitation. The software includes videogames with biofeedback for training at home which could increase the patient’s adherence to the treatment [23]. The new device promotes patient independence in the rehabilitation process and reduces social and health care costs [24]. This new instrument, unlike the others developed up to now, proposes accurate assessments and future treatments based on gamification (Table 1). Moreover, compared to current tongue force instruments, this new device has already demonstrated good validity values and a high intrarater reliability, ensuring its safe use in the clinic [25]. Nevertheless, as a first step in the validation process, good inter-rater reliability for this device is also needed. Reliability is defined as the probability that a system, instrument, or device could perform a specific function in certain circumstances. It refers not only to the agreement but also the consistency between measurements. Moreover, random and systematic errors are needed to obtain reliability data and ensure accurate results. For this reason, devices must demonstrate a good stability and reliability before their use or commercialization. This makes the device safer during its use in a variety of clinical and research settings as well as by any type of person (professionals, patients, or patients’ relatives) and ensures the security to be used with patients and different environmental conditions. According to this, it is established that this type of study should be developed in healthy subjects at first for trying to protect vulnerable individuals and could ensure that the device is safe for its condition. After that, reliability studies must be performed in patients for demonstrating the clinical usefulness [26].

The main objectives of this study were to determine the intra- and inter-rater reliability and the minimum detectable change (responsiveness) in the maximum tongue force measurements using a newly developed device. The authors of the study wanted to demonstrate that this device could measure with the same reliability independently of the professional or patient who is using it. Since current commercial systems do not have enough evidence of their validity or inter-rater reliability and due to the high costs of technologies such as fluoroscopy, currently applied screening techniques are very subjective and depend on the training and experience of the therapist. This makes the devices less reliable. For this reason, demonstrating the reliability and sensitivity of our system would help in developing more objective assessments, regardless of the therapist performing the measurements.

## 2. Materials and Methods

An intra- and inter-rater reliability single-blind study with repetitive measurements was conducted based on the guidelines for reporting reliability and agreement studies (GRRAS) [27]. This study was approved by the Ethics Committee from the Centro Superior de Estudios Universitarios La Salle (CSEULS) of the Universidad Autónoma de Madrid (project code: CSEULS-PI-036/2019). Subjects were recruited from the CSEULS of the Universidad Autónoma de Madrid. Participants were recruited through nonprobability sampling.

### 2.1. Subjects 

A total of 26 asymptomatic subjects older than 18 participated in this study. The sample size was calculated based on the intraclass correlation coefficient (ICC) values obtained in previous studies [28,29,30,31]. An ICC of 0.90 was estimated based on the hypothesis. A sample of 26 subjects with 2 measurements per subject was needed to achieve 80% power (β = 0.2) to detect an ICC of 0.90, with a significance level of 0.05.

Subjects were excluded if they presented TMD, cancer, or an active infection of the neck/head/mouth, had a history of orofacial or cervical surgery, had temporomandibular/orofacial/cervical acute pain before or during the test, were undergoing physical therapy for the neck or craniofacial region, had more than 6 points out of 10 on the subjective perception of fatigue scale, or had neurological disorders and rheumatic systemic disorders.

### 2.2. Instrumentation

**Figure 1:**
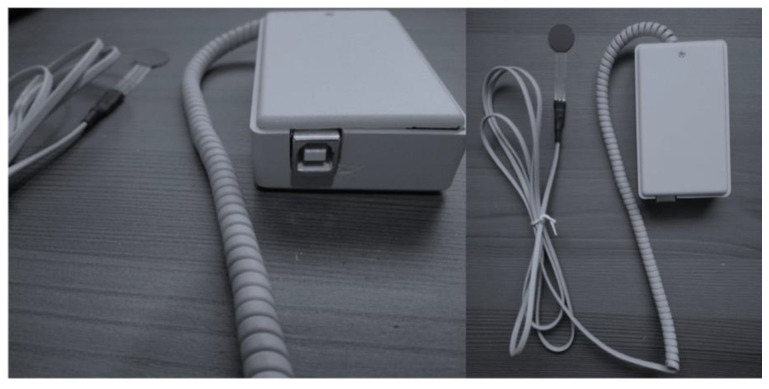
Prototype tongue force device (inferior and upper views). Small casing protecting the backplane, extralong cable, and piezoelectric sensor.

**Figure 2:**
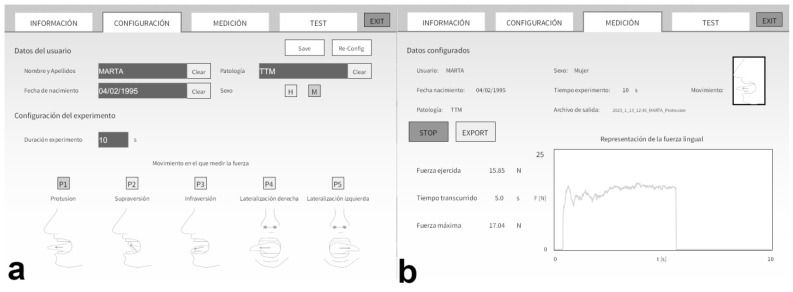
Spanish prototype device interface view. (**a**): Configuration screen view for the main characteristics and personal data of the patient, and the duration of the experiment; (**b**): Measurement screen view for the specific movement that is being measured with the time, maximum force, force exerted during each second, and a feedback representation of the force exerted.

The new low-cost prototype device, introduced in a previous article [23], was specifically designed and developed to measure tongue force objectively and accurately. The device consists of a physical part and associated software. The physical part consists of a hardware system that measures the pressure exerted on a piezoelectric sensor (FSR 402, Interlink Electronics Inc., Irvine, CA, USA) [32] and transmits the information with an Arduino UNO via a wired connection to a personal computer, where the software is located (Figure 1). This type of sensor is a very thin and flexible piezoelectric that does not cause any discomfort to the patient. The software is responsible for processing and displaying the information in real time. In addition, the software facilitates the recording of the demographic information of the subjects and the information recorded by the sensor is stored in a database for the subsequent extraction of reports (Figure 2).

The interface has a user-centered design for ease of use in the clinical environment. The device can measure the pressure exerted on the sensor by placing it in different positions. Depending on the positioning of the sensor, it is possible to measure the force exerted in the following movements: lip to lip, tongue elevation (tongue against the anterior part of the hard palate), tongue depression (tongue against the jaw), right tongue lateralization (tongue against the right cheek), left tongue lateralization (tongue against the left cheek), and their combinations.

### 2.3. Procedure

Two experienced physical therapists with more than 3 years’ experience working in the cervico-craniofacial area were trained on how to perform the maximum tongue force test and the whole intervention. The biomedical engineer that developed the device specifically helped and trained both physical therapists on how to use the new device. The tongue force test was performed on each participant in a sitting position for the tongue movements mentioned above in Section 2.2. Two measurements of each tongue movement were performed by each rater. The GraphPad Quickcals website was used to randomize which assessor had to go first on the measurements. The measurements were performed on the same day for both raters. Each rater was blind to the other rater’s measurements. The subjects and raters were not able to see the results between the 2 measurements performed for each movement.

**Figure 3:**
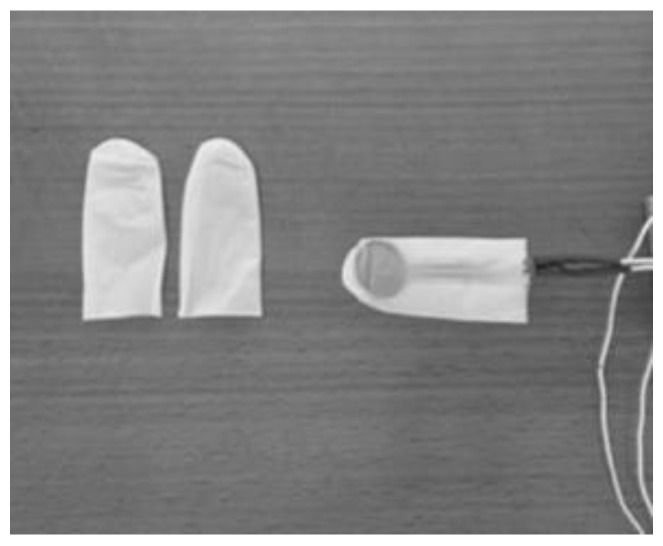
Single-use hypoallergenic protection covering the whole sensor to protect it.

**Figure 4:**
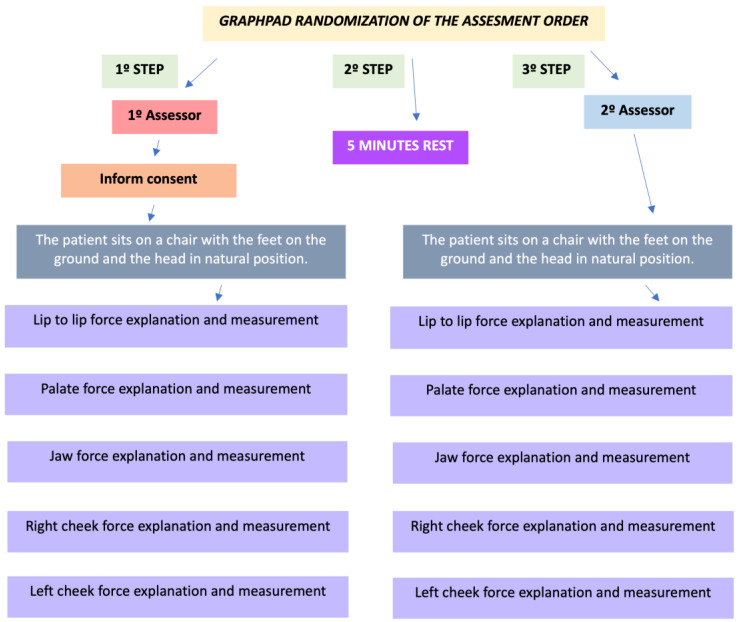
Descriptive graphic of the procedure.

A single-use hypoallergenic protective measure made of nitrile was used to cover the sensor during the measurements for each subject (Figure 3). The single-use protection was not changed during the whole test, only between different participants. The subjects were asked to sit with their back against the chair, feet on the ground, and head in its natural position. The tongue sensors were placed by the subjects following the instructions given by the rater according to the movement tested. During the maximum tongue force test, the subjects were then asked to exert the maximum tongue force against the sensor for 10 s. A 5 min resting period was used between each measurement. Firstly, for the lip-to-lip movement, the sensor was placed between the lips, not including the teeth. Secondly, the sensor was placed behind the superior incisors in the anterior part of the hard palate for the tongue elevation movement. Thirdly, the subjects placed the sensor behind the inferior incisors in the jaw for the tongue depression movement. Finally, right and left tongue lateralization movements were developed by placing the sensor in the anterior part of the right and left cheeks, respectively. The whole procedure is described in Figure 4.

### 2.4. Analysis and Sample Size

The sample size was calculated based on the intraclass correlation coefficient (ICC) values obtained in previous studies [28,29,30,31]. An ICC of 0.90 was estimated based on the hypothesis. A sample of 26 subjects with 2 measurements per subject was needed to achieve 80% power (β = 0.2) to detect an ICC of 0.90, with a significance level of 0.05.

The interclass correlation coefficient and standard error of measurement (SEM) were used to calculate the reliability. The ICC_3,1_ was designated as the two-way analysis of variance mixed model for the absolute agreement of single measures. The ICC_3,2_ was designated the same way as the ICC_3,1_ but using the average of the two measures of each rater to determine the inter-rater reliability [33]. Intraclass correlation coefficient values greater than 0.75 indicate good reliability, those between 0.50 and 0.75 indicate moderate agreement, and those below 0.50 indicate poor agreement [33]. A 95% confidence interval (CI) was also calculated, and *p* < 0.05 was used as the level of statistical significance.

Bland–Altman plots were constructed using mean differences between measurements [34]. Limits of agreement (LOA) were calculated as mean differences ± (standard deviation multiplied by 1.96) [35]. Calculation of the occurrence of systematic or random changes in the data means that it was performed through a calculation of 95% confidence intervals (CI) of the mean differences between the values of the measurements.

The responsiveness was determined with minimal detectable change at 90%, which was calculated as SEM × 1.65 × √2 [36,37]. The MDC_90_ expresses the minimal change required to be 90% confident that the change observed between two measurements reflects a real change (sensitive measure) and not a measurement error.

## 3. Results

A total of 26 subjects were included in the reliability analysis (57.7% men and 42.3% women). The average age of the sample was 25.69 years old with a standard deviation of 7.46 years old. In relation to body mass index, it was 26.1 (25.7–26.5; 95%CI) in men and 24.1 (23.7–24.7; 95%CI) in women. In addition, the percentage of participants with or in the process of completing tertiary education was 63%. According to the Shapiro–Wilk test, the data were normally distributed (*p* > 0.05).

### 3.1. Intrarater Reliability Results

The descriptive data for intrarater reliability, ICC_3,1_, SEM, MDC_90_, and Bland–Altman analysis with the 95%CI and LOA are summarized in Table 2. Good-to-excellent intrarater reliability for all tongue movements was found for both raters (ICC_3,1_ ≥ 0.80). The SEM was <0.70 for rater A and <0.98 for rater B. The MDC was between 1.10 and 1.64 for rater A and between 0.96 and 2.30 for rater B.

### 3.2. Inter-Rater Reliability Results

**Figure 5:**
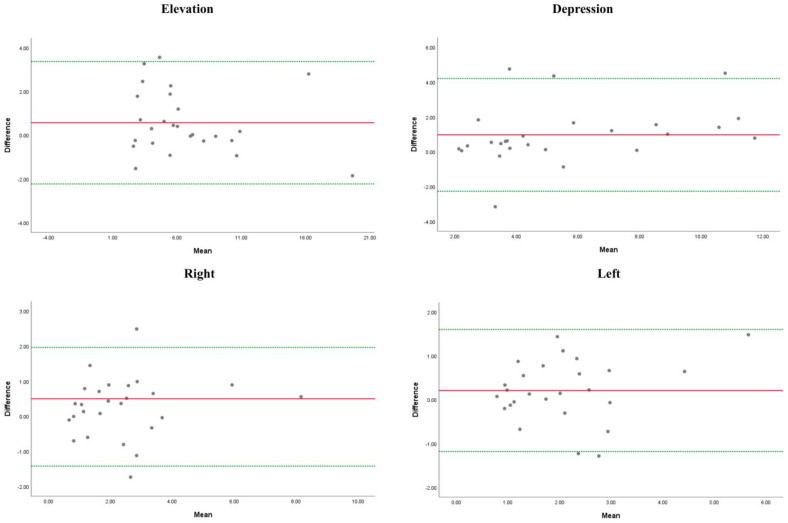
Graphical representations of the Bland–Altman plot. The red line is the mean difference. The green lines represent the Limits of Agreement (LOA).

The descriptive data for inter-rater reliability, ICC_3,2_, SEM, MDC, and Bland–Altman analysis with the 95%CI and LOA are summarized in Table 3. Good-to-excellent intrarater reliability for all tongue movements was found for both raters (ICC_3,2_ ≥ 0.80). The SEM was <1.29. The MDC was between 1.20 and 3.01. Graphical representations of the Bland–Altman plot are shown in Figure 5.

## 4. Discussion

As far as the authors know, this is the first study evaluating the maximum tongue force in four different directions of tongue movement. According to the results, a good-to-excellent intra- and inter-rater reliability was found for all movements. The measurements were also responsive to detect real changes.

This was also the first study testing the reliability of a device with a force-sensitive resistor (FSR) sensor to measure the maximum tongue force. Although the MOST device is composed of the same type of sensor, its reliability has not been tested [20]. There is currently no gold standard for maximum tongue force outcome measurements. That is why the results from this study are compared with the devices that are often used in clinical practice and research.

The present study has demonstrated an excellent intrarater reliability for maximum tongue force measurements of the superior, inferior, and right tongue movements (ICC_3,1_ > 0.93) and a good intrarater reliability for measurements of the left tongue movement (ICC_3,1_ > 0.82). The measurements of the superior tongue movement obtained the highest ICC_3,1_ values (>0.95). These values were slightly greater than those found for the reliability measurements of tongue force in superior movements using the IOPI device, which ranged from 0.77 to 0.90 [38]. Likewise, better ICC values were obtained when compared to the study by White et al., who reported an excellent intrarater reliability for the KSW device in a healthy population (ICC = 0.92) [19]. In reference to the Oropress reliability results, similar ICC values were found (ICC = 0.86) when compared to the present study [17].

An excellent inter-rater reliability for measurements of the tongue force in elevation (ICC_3,2_ = 0.94) and a good inter-rater reliability for measurements of the tongue force in depression and right and left lateralization (ICC_s3,1_ = 0.83, 0.87 and 0.81, respectively) were found in the current study. Youmans and Stierwalt (2006) obtained a 94% inter-rater agreement (r = 0.94) during the maximum isometric force measurement using the IOPI device [39]. The IOPI device is commonly used; however, it is only used to measure tongue force in one direction (tongue elevation). Additionally, the IOPI analysis protocols are different from the ones utilized in the present study. While the common IOPI protocol for analysis uses the highest value obtained during the three tests or the mean of the two best tests, the current study used the mean of the two measurements. Nevertheless, researchers cannot define the analysis with any of the devices since there is no defined protocol. Similarly to the IOPI device, the KSW instrument only measures superior tongue force movement and commonly collects the higher measure of the three tests performed.

The inter-rater reliability of tongue force measurements using the IOPI device in subjects with different conditions was reported to be good to excellent (ICC > 0.75) [22], with the exception of a study evaluating dysarthria patients in which a moderate reliability was found (ICC = 0.535) [22,38]. However, there are no recent studies available on the evaluation of the inter-rater reliability for the IOPI in healthy subjects, and the authors of this paper believe that this should be the first step prior to measurement and use in patients. Likewise, there is no inter-rater reliability research for measuring tongue force with the KSW device. The KSW device uses the same type of sensor as the IOPI, a silicon air-filled bulb. The main difference is that the KSW bulb is fixed to the palate, providing more stability and reliability. Probably due to its multiple functions, the KSW device is used more for research evaluating tongue force during swallowing. According to Fei et al., the KSW device is more reliable than the IOPI when evaluating tongue force during the function of swallowing [40].

Regarding responsiveness, the SEM values were low for elevation, depression, and right and left tongue movements (1.03, 1.09, 0.60, and 0.51, respectively). The MDC values were also low for elevation, depression, and right and left tongue movements: 2.40, 3.01, 1.43, and 1.20, respectively. Therefore, the new device was able to capture real change in tongue measures in all directions. Although we can assure good reliability and responsiveness for the device presented in this study in an asymptomatic population, we cannot guarantee the same findings in symptomatic subjects yet. Only one previous study determined the SEM and the MDC of the IOPI device in asymptomatic subjects [38]. This study estimated these values using standard deviation (SD), while the present study based the calculation on the root mean square (RMS) [40]. The SD was used to estimate the SEM, avoiding possible uncertainties due to the selected ICC type [35]. Therefore, the evaluation of the SEM varies between studies. Additionally, a Bland–Altman method was used to evaluate agreement, including the LOA. A good LOA was found, and the SEM, MDC, and LOA revealed a good level of concordance. These values are very important for the use of the device in clinical practice as they ensure that any improvement in tongue force is due to the treatment rather than measurement errors.

This study demonstrated that the newly developed tongue force device is reliable for measuring the maximum tongue force in different directions within and in between professionals. The new device overcomes some limitations from the tongue devices commonly used in the literature. This validated, safe, portable, and easy-to-use device can allow patients to perform tongue exercises at home, and the ability of the device to display the tongue activity in real time may increase their motivation to progress with their rehabilitation program. All these features add to the fact that it is a low-cost instrument. We recommend that future studies are needed to test the tongue force device including both healthy subjects and patients. Additionally, future studies must include in silico/computational simulation to ensure that the force data used from the device is accommodated correctly [41].

### 4.1. Limitations 

This study presents some limitations. Nonprobability sampling is always a limitation of a study. Ideally, a sufficiently large population would have been accessible for probability sampling. The reliability of the developed tongue force device was tested on healthy young subjects mainly (at an average of 25.7 years of age) and, therefore, these results should be taken with caution when transferring them to other populations. Further studies should test the device in different age groups in order to generalize the results. Likewise, future studies should include subjects with different health conditions. The results showed a significant statistical difference in some values of the Bland–Altman plot. These differences are close to 0 and all mean difference values are below the MCD in all cases. This led us to assume those results are statistically significant but not clinically relevant. The minimal clinically important difference (MCID) should be evaluated in future studies. Likewise, the values of other populations must be established and validated in future studies as with any measurement device or questionnaire.

### 4.2. Clinical Implications

From a neurophysiological point of view, it is known that the cerebral cortex has areas where information (input and output) from the V (trigeminal nerve), VII (facial nerve), and XII (hypoglossal nerve) cranial nerves is integrated [42]. In this way, these cranial nerves control the muscles of mastication, facial gestures, and the tongue, respectively, in order to achieve the optimal functionality of the entire system during speech and mastication, among other functions. Additionally, we have already published an observational study which showed significant differences in the maximum tongue force between asymptomatic women and those with chronic temporomandibular disorder, corroborating the necessity for the assessment of the tongue force in this pathology [43]. In this article, a decrease in tongue strength of about 30% on average across all directions was found in the group of patients with chronic TMD. In line with this, clinical experience shows that many patients with TMD (especially the chronic type) have lingual alternations both in terms of flexibility (length) and strength in various directions.

This new device to measure tongue force allows obtaining objective measurements of tongue force in clinical practice in order to help clinicians with the diagnosis process and treatment progression. This will give clinicians and patients real data to observe the changes during the treatment. Moreover, the new tongue force device has a diagnostic interface and treatment interface with different games to train the force at home and in the clinic. This training with games will motivate the patients and increase the adherence to the treatment. This offers an accessible device for patients and clinicians due to the fact that the few that are available in the market have this limitation and are much more expensive. Moreover, its validity has been proved in a previous study that has been recently published [25]. This could be a new tool to consider and add for the assessment and treatment of these patients. Likewise, as a new tool in the treatment of TMD, it could decrease the sociosanitary costs that this pathology implies for the sanitary system due to its chronicity.

## 5. Conclusions

This study showed a good-to-excellent intra- and inter-reliability for the newly developed device to measure the maximum tongue force in four different directions in an asymptomatic population. The measurements with the new device were also able to detect real changes, suggesting a more sensitive measure (good responsiveness in the device). These results confirmed that the device is suitable for objective and precise tongue measurements independently of the subject that is using this tool. The new prototype device seems to be an improved tongue force measurement tool that is safe, validated, and more accessible than others on the market.

## Figures and Tables

**Table 1:** Feature comparison between the new device, the IOPI, the KSW, and the MOST instruments.

	Easy Portability	Individualized Exercises	Visual Feedback and Videogames	Home Training	Patient Follow-Up	Price of the Device
NEW DEVICE	X	X	X	X	X	EUR 42,544
IOPI	X			X		EUR 800–2000
KSW						Not available
MOST	X					Not available
OROPRESS	X		X (feedback only)			Not available

**Table 2:** Intrarater reliability (*n* = 26).

Outcome Measurements (Newtons)	Mean ± SD	Mean ± SD			
	1st Measure	2nd Measure	ICC (95%CI)	SEM	MDC 90%
Rater A					
Elevation	6.92 ± 4.26	6.87 ± 3.94	0.97 (0.93–0.99)	0.70	1.64
Depression	6.02 ± 3.39	6.09 ± 3.43	0.96 (0.91–0.98)	0.68	1.58
Right	2.58 ± 1.77	2.45 ± 1.84	0.93 (0.85–0.97)	0.47	1.10
Left	2.11 ± 1.21	2.25 ± 1.40	0.84 (0.68–0.92)	0.52	1.21
Rater B					
Elevation	6.19 ± 4.78	6.46 ± 4.07	0.95 (0.89–0.98)	0.98	2.30
Depression	5.17 ± 2.91	5.00 ± 2.87	0.94 (0.88–0.98)	0.70	1.64
Right	2.12 ± 1.69	2.37 ± 1.69	0.94 (0.87–0.97)	0.41	0.96
Left	1.83 ± 1.09	2.10 ± 1.22	0.82 (0.65–0.92)	0.49	1.14
		Bland–Altman			
		Mean difference ± SD	95%CI	LOA (Inf-Sup)	
Rater A					
Elevation		0.06 ± 1.05	(−0.34 to 0.46)	(−2.00 to 2.12)	
Depression		−0.07 ± 0.98	(−0.45 to 0.31)	(−2.00 to 1.85)	
Right		0.13 ± 0.68	(−0.13 to 0.39)	(−1.20 to 1.46)	
Left		−0.14 ± 0.73	(−0.42 to 0.14)	(−1.57 to 1.29)	
Rater B					
Elevation		−0.27 ± 1.42	(−0.82 to 0.28)	(−3.05 to 2.51)	
Depression		0.16 ± 0.97	(−0.21 to 0.53)	(−1.74 to 2.06)	
Right		−0.24 ± 0.60	(−0.47 to −0.01)	(−1.42 to 0.94)	
Left		−0.27 ± 0.69	(−0.54 to −0.005)	(−1.62 to 1.08)	

Abbreviations: ICC: intraclass correlation coefficient; CI: confidence interval; SEM: standard error of measurement; MDC: minimum detectable change; SD: standard deviation; LOA: limits of agreement.

**Table 3:** Inter-rater reliability (*n* = 26).

Outcome Measurements (in Newtons)	Mean ± SD	Mean ± SD			
	Rater A	Rater B	ICC (95%CI)	SEM	MDC 90%
Elevation	6.89 ± 4.07	6.32 ± 4.38	0.94 (0.86–0.97)	1.03	2.40
Depression	6.05 ± 3.37	5.08 ± 2.85	0.83 (0.57–0.93)	1.29	3.01
Right	2.52 ± 1.77	2.24 ± 1.66	0.87 (0.73–0.94)	0.61	1.43
Left	2.18 ± 1.26	1.97 ± 1.10	0.81 (0.63–0.91)	0.51	1.20
	Bland–Altman				
	Mean difference ± SD	95%CI	LOA (Inf-Sup)		
Elevation	0.57 ± 1.43	(0.02 to 1.12)	(−2.23 to 3.37)		
Depression	0.97 ± 1.65	(0.33 to 1.60)	(−2.26 to 4.20)		
Right	0.27 ± 0.86	(−0.06 to 0.60)	(−1.42 to 1.96)		
Left	0.21 ± 0.71	(−0.06 to 0.48)	(−1.18 to 1.60)		

Abbreviations: ICC: intraclass correlation coefficient; CI: confidence interval; SEM: standard error of measurement; MDC: minimum detectable change; SD: standard deviation; LOA: limits of agreement.

## Data Availability

Not applicable.
